# Supplementary material for: Time-restricted feeding reduced blood pressure and improved cardiac structure and function by regulating both circulating and local renin-angiotensin systems in spontaneously hypertensive rat model
Source: PLoS One. 2025 Apr 3;20(4):e0321078. doi: 10.1371/journal.pone.0321078 (PMC11967951; doi:10.1371/journal.pone.0321078)
Supplement: Attachment — Submitted filename: Response to Reviewers.docx [file pone.0321078.s003.docx]

Dear Editor and Reviewers,

Manuscript Title: Time-Restricted Feeding Reduced Blood Pressure and Improved Cardiac Structure and Function by Regulating Both Circulating and Local Renin-Angiotensin Systems in Spontaneously Hypertensive Rat Model
Manuscript ID: PONE-D-24-49902

We sincerely thank you, the editor, and the reviewers for their constructive comments and suggestions regarding our manuscript. We have carefully considered each point raised and have made revisions accordingly to improve the quality of the manuscript. Below, we provide a detailed response to each comment. To facilitate the review process, we have structured our responses as follows:

1. Reviewer comments are presented in **bold**.
2. Our responses follow immediately in plain text.
3. All modifications in the manuscript are marked in red font, so that the reviewers can easily identify the changes.

Response to Reviewer 1

Comment 1: **Input some statistic figure for the hypertension as global health issue.**
Response: : I have added some statistical data regarding hypertension as a global health issue in the Introduction section. Please refer to lines 28-32 in the revised manuscript.

Comment 2: **IF stands for what?, abbreviation should be put at the first time when it is used.**
Response: IF stands for intermittent fasting, and its full term has been specified at its first use. Please refer to line 38 in the revised manuscript.

Comment 3: **In text, it mentioned, “RAS is a central component of the pathological process of hypertension and hypertension-induced cardiac remodeling and is a key therapeutic target”… Include some pathological evidence on RAS that related to hypertension after line 50.**
Response: I have added some pathological evidence related to RAS and hypertension. Please refer to lines 54-61 in the revised manuscript.

Comment 4: **For the laboratory animals, authors should present the animal sampling, or sample size calculation, inclusion and exclusion criteria, and or be included as supplementary files.**
Response: I have added the methods for Laboratory Animals selection, inclusion and exclusion criteria, as well as details of the animal housing environment. Please refer to lines 79-87 in the revised manuscript.

Comment 5: **Please add the citation to support the sentences at line 214-216 regarding the SHI’ S study of ADF intervention.**
Response: I have added the citation. Please refer to line 234 in the revised manuscript.

Comment 6: **Please add future perspective of the study.**

Response: I have added the future perspective of the study at the end of the Discussion section. Please refer to lines 296–309 in the revised manuscript.

Comment 7: **References are not properly written in the bibliography section as per journals criteria and style.**

Response: I have adjusted the format of all references in accordance with the journal's requirements. Please refer to the bibliography section in the revised manuscript.

Response to Reviewer 2

Comment 1: **the current statistical global update for hypertension and its associated comorbidities.**
Response: I have added global statistical data on hypertension and its cardiovascular comorbidities in the Introduction section. Please refer to lines 28–32 in the revised manuscript.

Comment 2: **All abbreviations must be defined first before being used in the text. Numerous terms are not explained properly while using abbreviations.**
Response: IF stands for intermittent fasting, and its full term has been specified at its first use. Please refer to line 38 in the revised manuscript.

Comment 3: **The mechanism of RAAS’ s pathological association with hypertension and cardiac remodeling needs further explanation with the help of recent literature for a better understanding of the hypothesis and objectives of the study.**
Response: I have added some pathological evidence related to RAS and hypertension, emphasizing the critical role of the RAS in the development and progression of hypertension. Please refer to lines 54–61 in the revised manuscript.

Comment 4&5: **4. Did the authors observe any inclusion and exclusion criteria for the selection of animals? SHRs belong to the genetic model of hypertension and are age-dependent, competing with the criteria for hypertension.**

**5. If yes, please also provide complete details for animal grouping animals, acclimatization period, and environment**
Response: Yes, I have added the methods for Laboratory Animals selection, inclusion and exclusion criteria, as well as details of the animal housing environment. Please refer to lines 79-87 in the revised manuscript.

Comment 6: **To improve the discussion further, compare the data findings with the recent literature on parallel lines to support the conclusions and study hypothesis.**
Response: I have incorporated recent literature related to intermittent fasting, hypertension, and the RAS into the discussion, comparing them to highlight their relevance in supporting the conclusions and study hypothesis. Please refer to lines 241, 266-269, and 278-287 in the revised manuscript.

Comment 7&8: **7. A few studies are not properly cited in the discussion part, which creates ambiguity while understanding the study findings.**

8. **An example is "in lines 221 to 223, there is no supporting evidence from previous work on, if any, for the limited efficiency of TRF in prolonged intervention.**
   Response: I have reorganized the relevant references and rewritten the paragraph corresponding to lines 221-223 in the initial draft to provide supporting evidence and eliminate ambiguity, ensuring clearer and more accurate expression.

Comment 9: **In conclusion, elaborate clearly that the study's outcome will assist in highlighting and identifying the therapeutic strategies for cardiac remodelling.**

Response: I have added the future perspective of the study and emphasized that the study's findings will assist in highlighting and identifying the therapeutic strategies for cardiac remodeling. Please refer to lines 296-309 in the revised manuscript.

Comment 10: **Some references are not properly cited. The journal editorial office must check the style of the references before final acceptance of the manuscript.**

Response: I have adjusted the format of all references in accordance with the journal's requirements. Please refer to the bibliography section in the revised manuscript.

Finally, we deeply appreciate the reviewers' and editor's valuable comments and constructive suggestions, which have significantly improved the quality of our manuscript. Your insightful feedback has helped us refine our study and present our findings in a clearer and more impactful manner.

If there are any further comments, questions, or concerns regarding the manuscript, please do not hesitate to contact us. We are more than willing to address them promptly and make additional revisions if necessary.

Thank you once again for your time, effort, and valuable contributions to our manuscript.
